# Serious Game–Based Attention Assessment for Attention-Deficit/Hyperactivity Disorder in Children and Adolescents: Preliminary Cross-Sectional Convergent Validation Study

**DOI:** 10.2196/80441

**Published:** 2026-08-28

**Authors:** Alejandro Panagiotidis-Arrizabalaga, Ana Lavalle, Miguel A Teruel, Javier Sanchis, Nicolás Ruiz-Robledillo, Borja Costa-López

**Affiliations:** 1 Department of Software and Computing Systems Polytechnic School University of Alicante Sant Vicent del Raspeig, Alicante Spain; 2 Alicante Institute for Health and Biomedical Research (ISABIAL) Alicante Spain; 3 Department of Health Psychology Faculty of Health Sciences University of Alicante Sant Vicent del Raspeig, Alicante Spain

**Keywords:** adolescents, assessment, Attention Robots, attention-deficit/hyperactivity disorder, children, impulsivity, neuropsychology, serious game

## Abstract

**Background:**

Attention-deficit/hyperactivity disorder (ADHD) is a common neurodevelopmental disorder in children, characterized by inattention, hyperactivity, and impulsivity that significantly disrupt daily functioning. Comprehensive ADHD assessment requires multimethod and multi-informant evaluation, and objective attention measures can complement this process. Serious game–based assessments may further support this process by combining structured task mechanics and dynamic audiovisual guidance in a child-oriented digital environment.

**Objective:**

This study aimed to develop and validate Attention Robots, a serious game designed for assessing behavioral attention indicators in children and adolescents. The goal was to examine whether its outputs corresponded with indicators from the widely used D2-R Test of Attention as preliminary evidence of convergent validity.

**Methods:**

In this preliminary cross-sectional convergent validation study, 58 participants (34 male and 24 female individuals) aged 6-18 years, recruited through clinical referral and community sources, completed Attention Robots followed by the D2-R test. The original group variable, assigned by the project psychologists, identified 19 participants in the experimental group and 39 participants in the control group. Attention Robots presents timed rows of robot stimuli and asks participants to identify and select predefined target robots. Spearman and Pearson correlations compared working speed, omissions, commissions, concentration, and precision across both instruments.

**Results:**

Primary cross-test correlations were positive. Spearman correlations were observed for working speed (ρ=0.905, 95% CI 0.84-0.94; *P*<.001), omissions (ρ=0.333, 95% CI 0.08-0.54; *P*=.01), commissions (ρ=0.496, 95% CI 0.27-0.67; *P*<.001), concentration (ρ=0.926, 95% CI 0.88-0.96; *P*<.001), and precision (ρ=0.607, 95% CI 0.41-0.75; *P*<.001). Pearson correlations showed a similar pattern for working speed (r=0.911, 95% CI 0.85-0.95; *P*<.001), omissions (r=0.278, 95% CI 0.02-0.50; *P*=.04), commissions (r=0.396, 95% CI 0.15-0.59; *P*=.002), concentration (r=0.922, 95% CI 0.87-0.95; *P*<.001), and precision (r=0.574, 95% CI 0.37-0.72; *P*<.001). These findings support preliminary convergent correspondence between corresponding behavioral indicators, but they do not establish interchangeability, discriminative validity, diagnostic use, or superior engagement or acceptability.

**Conclusions:**

Attention Robots provides preliminary evidence of convergent validity with the D2-R for behavioral attention indicators in children and adolescents. The innovative aspect of this study is the translation of D2-R–based attentional demands into a child-oriented serious game environment with robot-based stimuli, dynamic audiovisual guidance, and structured behavioral data capture. Unlike previous digital or serious game approaches, this study empirically compares game-derived indicators with corresponding indicators from an established attention test rather than evaluating only task feasibility, engagement, or intervention effects. This work contributes to the field by providing a preliminary validation of a serious game format that may support the collection of objective attention-related data in children and adolescents. In applied contexts, Attention Robots could be further developed as a complementary tool within multimethod ADHD assessment, although larger studies are required to evaluate subgroup performance, agreement, diagnostic use, and user experience before clinical adoption.

## Introduction

### Background

Attention-deficit/hyperactivity disorder (ADHD) is characterized by an enduring pattern of inattention, hyperactivity, and impulsivity that significantly disrupts functioning or development [1]. ADHD is estimated to affect approximately 5%-8% of children worldwide [2]. ADHD is also associated with measurable difficulties in sustained attention, inhibitory control, working memory, processing speed, and executive functioning [3,4]. Based on individual symptoms, three diagnostic categories delineate the types of ADHD: (1) predominantly inattentive ADHD, (2) predominantly hyperactive-impulsive ADHD, and (3) combined type ADHD [5].

According to the *DSM-5* (*Diagnostic and Statistical Manual of Mental Disorders, Fifth Edition*) [6], an ADHD diagnosis should be informed by the presence of symptoms that are frequent and persist for at least 6 months, typically manifesting between the ages of 3 and 6 years. Additionally, these symptoms should be observable across multiple settings, such as at home and at school. Consequently, it is essential to identify a significant functional impact across academic, social, and everyday life domains, and it requires a thorough evaluation of symptoms and impairments across these domains [7]. In this context, neuropsychological assessments offer objective measures that complement clinical observations and self-report measures [8], helping to confirm and specify the nature of cognitive deficits. For example, studies using tasks like the Go/No-Go or Stop-Signal Task have revealed impairments in inhibitory control among individuals with ADHD [9].

Building on the strengths of these objective methods, recent advances have introduced serious games as promising tools for both the assessment and treatment of ADHD [10,11]. Prior literature suggests that such systems may leverage interactivity and immersion to help maintain attention during evaluation [12], and can objectively capture real-time behavioral data. They have also been reported as potentially more enjoyable and less intimidating for some children than traditional formats [13].

In parallel with the development of serious games, there is a growing trend toward the use of AI-driven tools that leverage biometric data, such as electroencephalography, electrodermal activity (EDA), and eye-tracking, to support ADHD assessment and diagnosis [14]. These signals provide objective insight into attentional and cognitive states, and their integration is increasingly considered essential in modern digital interventions [15]. As a result, the incorporation of biometric data gathering into serious games has been proposed as a critical step toward enabling future AI-based applications in personalized ADHD detection and treatment [14].

### Related Work

In the realm of neuropsychological evaluation, ecological validity refers to how well an assessment reflects real-life situations [16]. Studies evaluating the ecological validity of traditional paper-and-pencil neuropsychological tests in both clinical and nonclinical populations have raised concerns about their limited ability to predict real-world functioning [17,18]. To address this, novel assessments have been developed to improve ecological validity. Some of these replicate traditional cognitive tasks [19], while others use technological systems to assess cognitive functioning [20].

Traditional neuropsychological tools remain central to the diagnosis of cognitive impairments associated with ADHD. However, technological advancements have enabled computer-based cognitive assessments that offer a precise and dynamic assessment of ADHD-related deficits [21,22]. For instance, in the study performed in [23], the authors introduced a game in which participants control a raccoon avatar that jumps over gaps, recording jump distances as a measure of attention. Another widely used digital assessment is the Conners’ Continuous Performance Test [18,24], which presents stimuli requiring quick responses, except when a specific “X-stimulus” appears, demanding inhibition. This test is based on the Go/No-Go paradigm [18,25], and multiple studies confirm its effectiveness in distinguishing individuals with ADHD from neurotypical controls [8]. These computerized tests facilitate precise measurement of sustained attention, working memory, and response inhibition [26], and are praised for their accuracy, adaptability, and standardized protocols [27,28].

Building upon these advances in adaptive cognitive assessments, recent systematic reviews have highlighted the growing role of AI-driven tools in improving ADHD diagnosis. In [29], the authors reviewed 98 studies from 2021 to 2024 and analyzed the application of AI models to biometric and behavioral data, such as electroencephalography, electrocardiogram, and magnetic resonance imaging data, as well as task performance data. Their findings emphasize the promise of physiological signals for objective diagnosis, while noting critical challenges, including the lack of standardized datasets, limited clinical adoption, and interpretability issues in deep learning models. Similarly, in [14], the authors conducted a scoping review focusing on unorthodox diagnostic approaches, including AI-enhanced gamification and real-time cognitive data collection. In parallel, the EPIDIA4Kids study [15] presents preliminary findings on the feasibility of using multimodal biometric data, including electroencephalography, eye tracking, and pupillometry, for noninvasive ADHD symptom screening in children.

Although recent studies have begun to demonstrate the potential of gamification for ADHD rehabilitation, there remains a gap in the development of serious games explicitly designed for ADHD detection and treatment [30]. Furthermore, most existing solutions lack validation against well-established standardized attention tests.

In this study, and consistent with the usage adopted in recent reviews of technology-driven interventions for ADHD [10], the term serious game refers to an interactive digital system designed for a purpose beyond entertainment, in this case the assessment of attentional performance. Within this broader framing, gamification refers more specifically to the incorporation of selected game-like elements into the assessment process. Attention Robots is therefore presented as a serious game for assessment, while incorporating a limited set of gamified interaction elements rather than reward-driven entertainment mechanics.

### Objective

In light of these considerations, this study presents Attention Robots, a serious game designed to quantitatively assess user attention. Although grounded in the principles of the D2-R Test of Attention [31], Attention Robots is not a direct digital transcription of the paper format. Instead, it introduces a computer-based interactive environment with robot-based stimuli, dynamic audiovisual instructions, row highlighting, auditory row-transition cues, and multimodal data acquisition. The robot theme was chosen by the psychology and design team as a developmentally appropriate visual format for children and adolescents: it was intended to be nonthreatening for younger participants while also avoiding an overly childish appearance for older ones. This design also provided a flexible visual framework for implementing rule-based task mechanics while preserving core attentional demands. More specifically, the current version of Attention Robots combines a serious game framework with selected gamified interaction elements, as noted earlier, but it was designed as a structured assessment environment rather than as a reward-based or progression-based entertainment game.

To examine preliminary convergent validity, we conducted a controlled study with 58 participants and compared the behavioral indicators obtained from Attention Robots with those obtained from the D2-R test. The analyses focused on cross-instrument correspondence rather than diagnostic classification, agreement, or user-experience superiority.

## Methods

### Research Design Overview

This study was designed as a preliminary cross-sectional convergent validation study. The aim was to examine the association between behavioral indicators obtained from Attention Robots and the same indicators obtained from the D2-R Test of Attention. The study did not include experimental manipulation, random assignment, diagnostic classification analysis, or longitudinal follow-up. The authorized ethical protocol dictated the specific order in which both instruments were administered: Attention Robots was administered first, and the D2-R test was administered in a second session. Therefore, rather than being a randomized experiment, crossover research, or diagnostic accuracy study, the study should be interpreted as a behavioral cross-instrument validation study.

### Participant Characteristics

The dataset comprises a total of 58 participants, with 34 (59%) male and 24 (41%) female individuals. Participants’ ages range from 6 to 18 years, covering a wide developmental span from early childhood to late adolescence. The average age of the participants is approximately 13.4 (SD 3.90) years. Based on the original demographic group variable assigned by the project psychologists, 19 (33%) participants were classified in the experimental group and 39 (67%) participants in the control group. In this variable, experimental participants were coded as 1 and control participants as 2. Within the experimental group, 17 (89%) participants were male and 2 (11%) were female, whereas the control group comprised 17 (44%) male and 22 (56%) female participants. Sex and group were taken from the original demographic source, and the group variable was used for descriptive reporting only, as all validation analyses were conducted in the pooled sample. This broad age range was retained to examine whether the behavioral indicators obtained from Attention Robots showed correspondence with those of the reference attention assessment across the intended population, rather than to establish age-specific normative performance.

### Sampling Procedures

Participants diagnosed with ADHD were referred by a specialized neurodevelopmental disorders unit within the Child and Adolescent Mental Health Service at General University Hospital Dr Balmis in Alicante, Spain. Diagnoses were established by licensed clinical psychologists from this specialized neurodevelopmental disorders unit based on clinical interviews with the patients, their families, and teachers, as well as structured assessments, following *DSM-5* criteria. Healthy control participants were recruited through personal contacts and recommendations from study participants and staff. Therefore, the study used a convenience-based recruitment approach, with ADHD participants recruited from a clinical referral setting and control participants recruited from the local community.

### Inclusion and Exclusion Criteria

Participants were included in the analysis if they were aged 6-18 years, had documented parental permission before participation, and completed both assessment sessions. Exclusion criteria included cooccurring mental conditions that could affect cognitive performance, such as intellectual disabilities or autism spectrum disorders, as well as the use of medication that could affect attention during the assessment.

### Sample Size, Power, and Precision

This was a feasibility/convenience sample for an initial validation study. No a priori power analysis was performed; therefore, effect estimates are interpreted with 95% CIs to indicate statistical precision. The sample size should be understood as supporting preliminary cross-instrument validation, rather than as a basis for drawing specific normative conclusions based on age, sex, or group.

### Ethical Considerations

**Figure 1:**
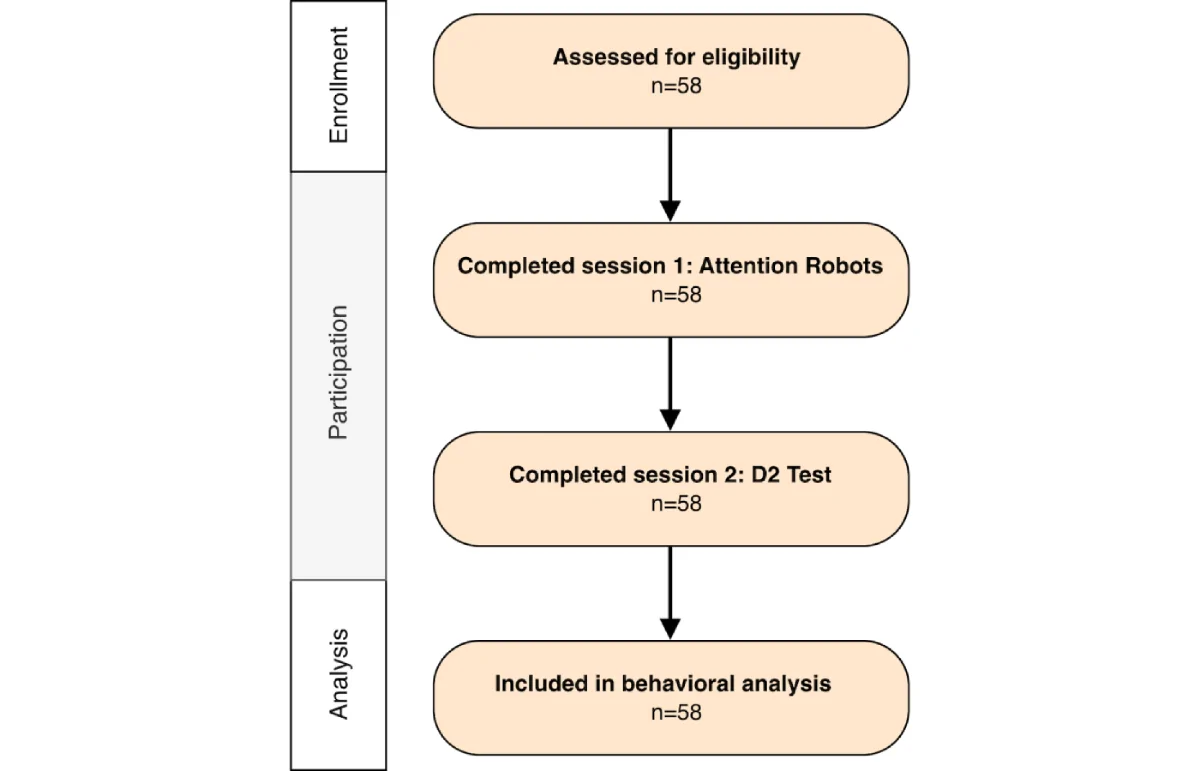
Participant flow for the analysis study sample.

Prior to initiating data collection, ethical approval was obtained from the Hospital Ethics Committee of the Alicante Institute for Health and Biomedical Research (ISABIAL; PI2022-139) and the Ethics Committee of the University of Alicante (UA-2023-05-17_1). This procedure was required because the data were collected from participants between the ages of 6 and 18 years. Before the assessment sessions, the psychologist responsible for the study met with the parents or legal guardians and the child to explain the study procedures, including the purpose of the study, the 2 assessment sessions, the tasks to be completed, the approximate duration of the sessions, the voluntary nature of participation, and the handling of participant data. Parents or legal guardians were given the written informed consent document to read and sign before the child participated in the study. Children also received an age-appropriate verbal explanation of the sessions, and the psychologist confirmed that they understood what they would be asked to do before starting the assessments. Each participant was assigned a unique study code at enrollment. The research dataset used for analysis did not contain directly identifiable information, and access to study data was restricted to the clinical psychology team responsible for participant recruitment and follow-up. The flow of participants included in the study is summarized in Figure 1. No financial compensation was provided to participants. All figures and supplementary materials do not contain identifiable images of individual participants.

### Instruments and Measures

#### Attention Robots

Attention Robots was developed as part of the Attention Game Series [32] to provide a structured digital task for capturing behavioral indicators of attentional performance in children and adolescents. In this study, it was evaluated as a complementary assessment format and not as a stand-alone diagnostic instrument or longitudinal monitoring tool. Although the game format was designed to be child-oriented, user-experience outcomes such as enjoyment, immersion, acceptability, and preference were not measured in this study.

Furthermore, the aforementioned game series will incorporate an array of devices designed to collect biometric data from players, encompassing a diverse range of physiological markers such as cerebral activity, electrodermal responses, cardiac rhythm, ocular tracking, among others.

**Figure 2:**
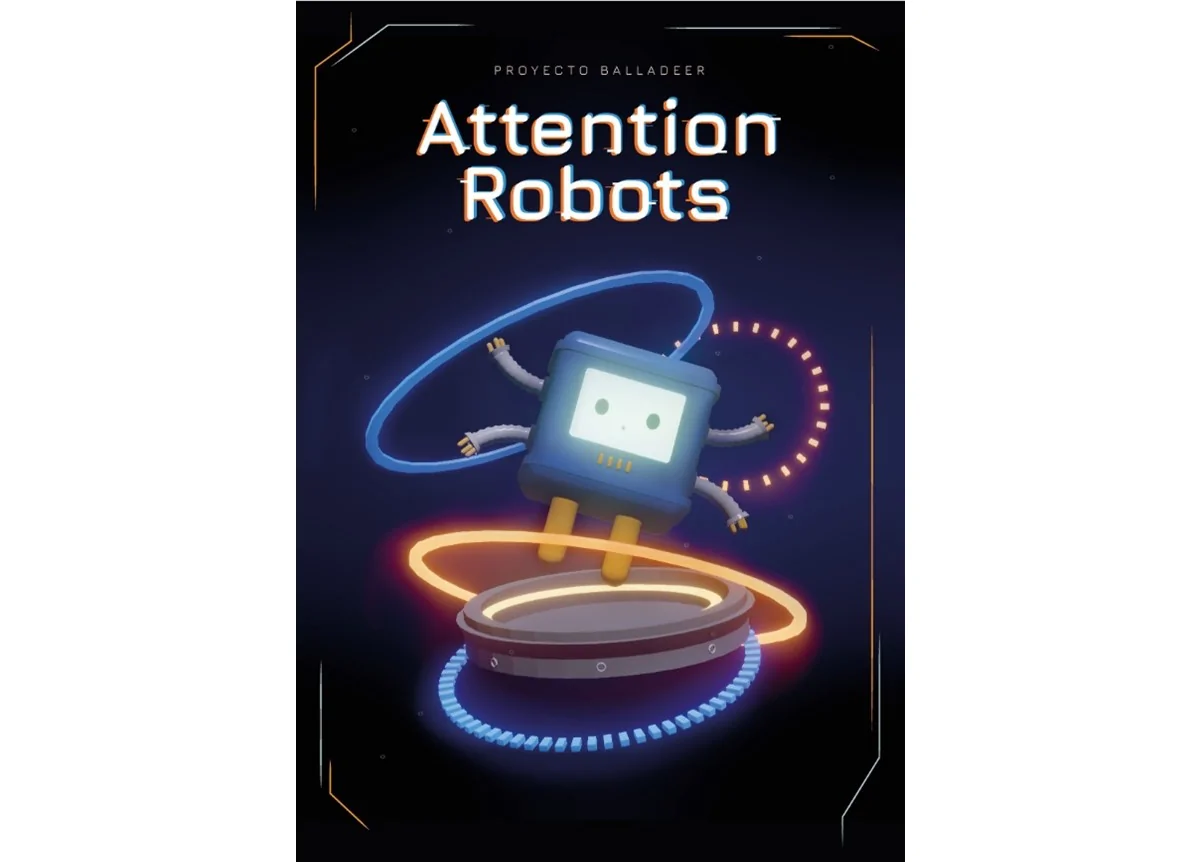
Cover screen of Attention Robots.

The integration of biometric data with performance metrics is intended to support future research on multimodal ADHD-related screening and monitoring. However, these uses require further validation and are not evaluated in this paper. In this context, Attention Robots represents the second serious game of the Attention series. Figure 2 shows the game’s start screen, and the game binaries are available in [32]. The game was developed under the guidance and expertise of a cadre of academic professionals specialized in psychology.

In this scenario, the player is presented with an array of diverse robots, each distinguished by unique attributes. The player’s objective is to identify and mark the robots that meet certain criteria, such as having precisely 2 arms, whether they are both on one side or one on each side, and remaining upright. This task assesses the player’s ability to pay attention to specific robot features according to the given rules.

The robot theme was selected because it provided an age-flexible and nonthreatening visual context; it was intended to be accessible for younger participants without appearing overly childish to older adolescents. Methodologically, this choice also created a broader design space for implementing task mechanics and visual rules while maintaining the core demands of selective attention, sustained scanning, inhibitory control, and speed-accuracy balance.

**Figure 3:**
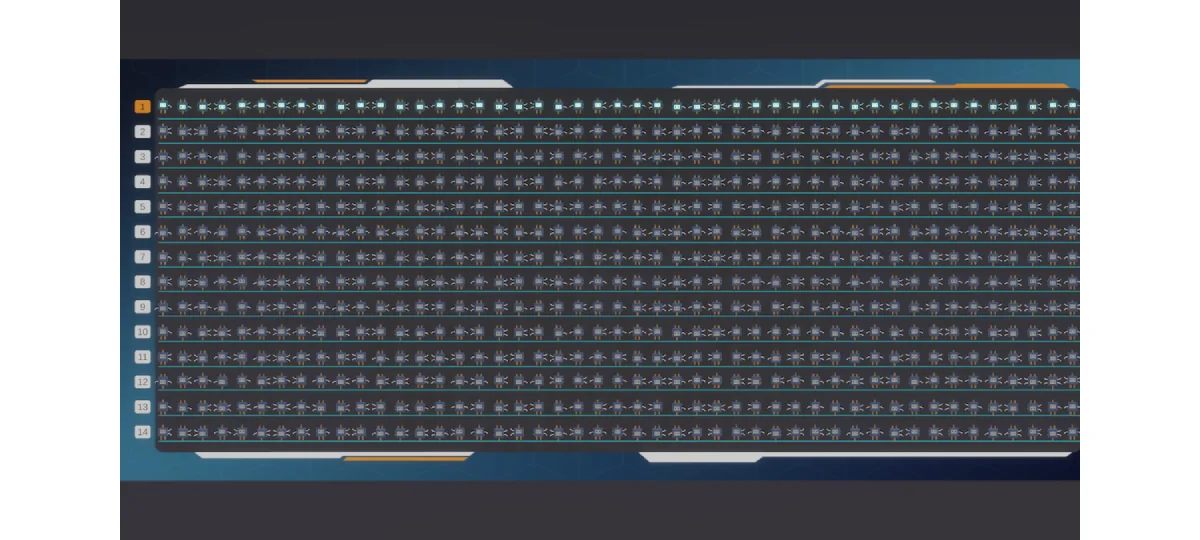
Attention Robots game grid.

As shown in Figure 3, the game is situated on a grid consisting of 14 rows, each of which contains 47 columns featuring different robot variations. This number of elements per row was selected to fit the computer display while preserving left-to-right visual scanning and the timed row-by-row structure, in consultation with the psychology team, and differs from the 57 elements per row of the D2-R. This extensive grid offers ample opportunities for players to assess their attention and decision-making abilities.

**Figure 4:**
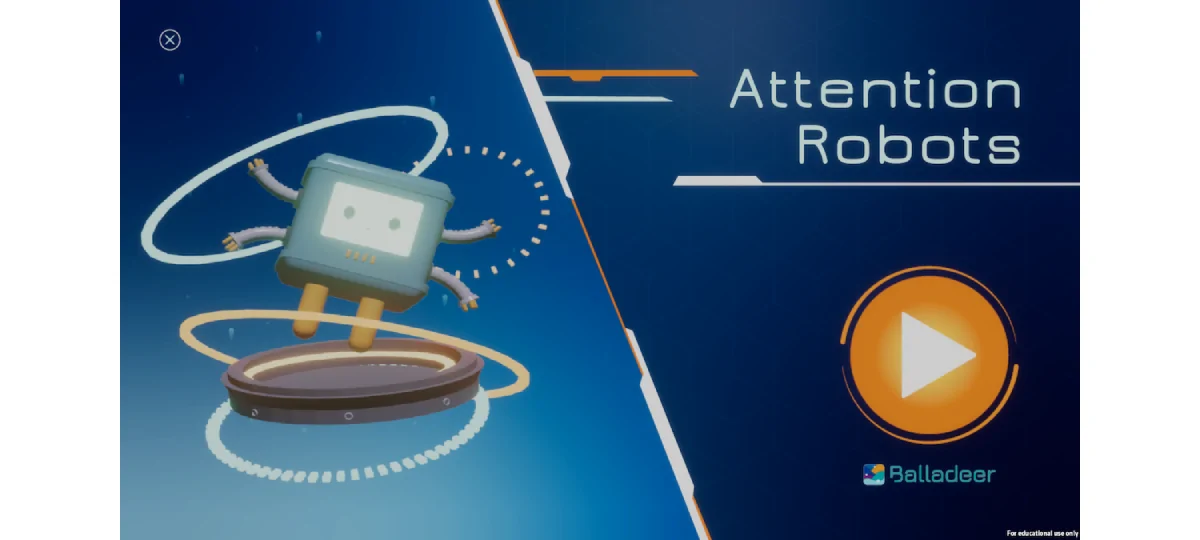
Main menu of Attention Robots.

The game begins with a structured process designed to facilitate player understanding and engagement. As shown in Figure 4, users first encounter the main menu, where they can start the game.

**Figure 5:**
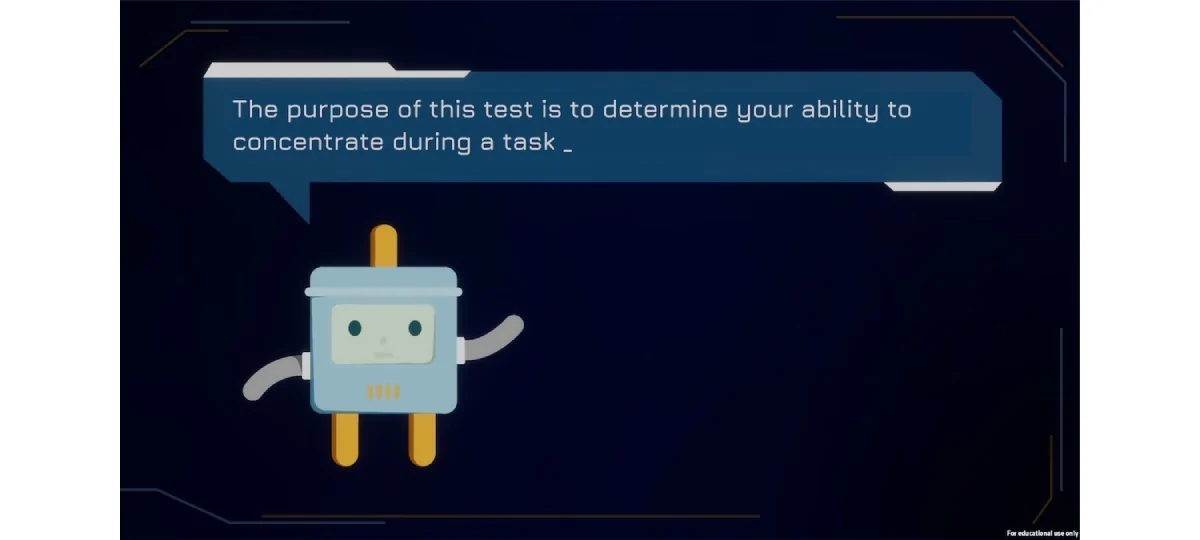
Attention Robots beginning of the tutorial.

Upon selecting the “start” option, a comprehensive video tutorial commences, providing both voice and written explanations of the game mechanics. This tutorial lasts approximately 2 minutes, ensuring that players are well-informed. In Figure 5, a screenshot of the beginning of the tutorial is shown.

**Figure 6:**
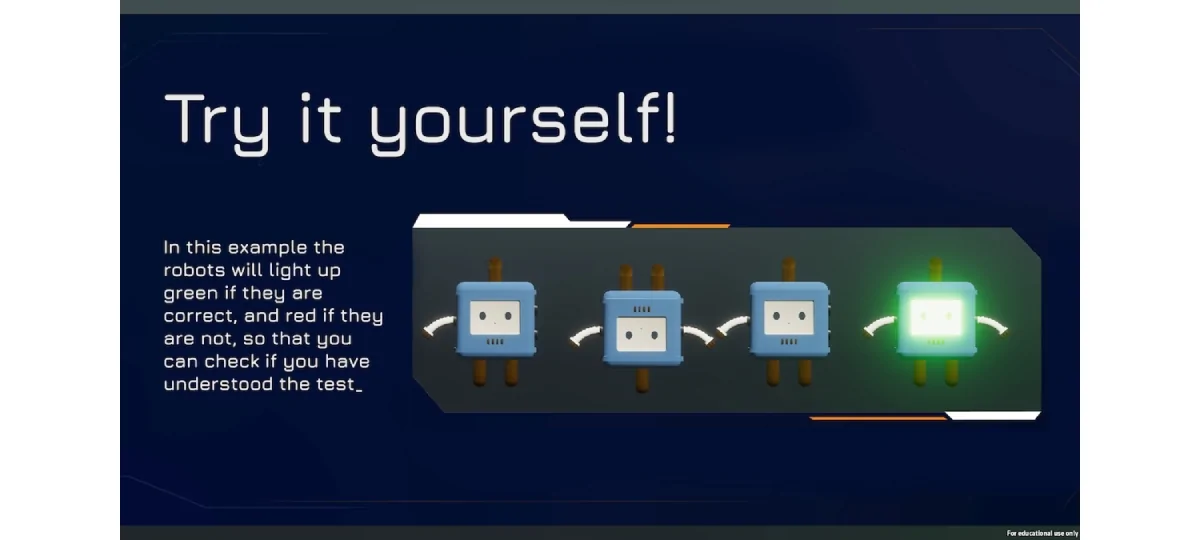
Attention Robots example: correct robot marked.

**Figure 7:**
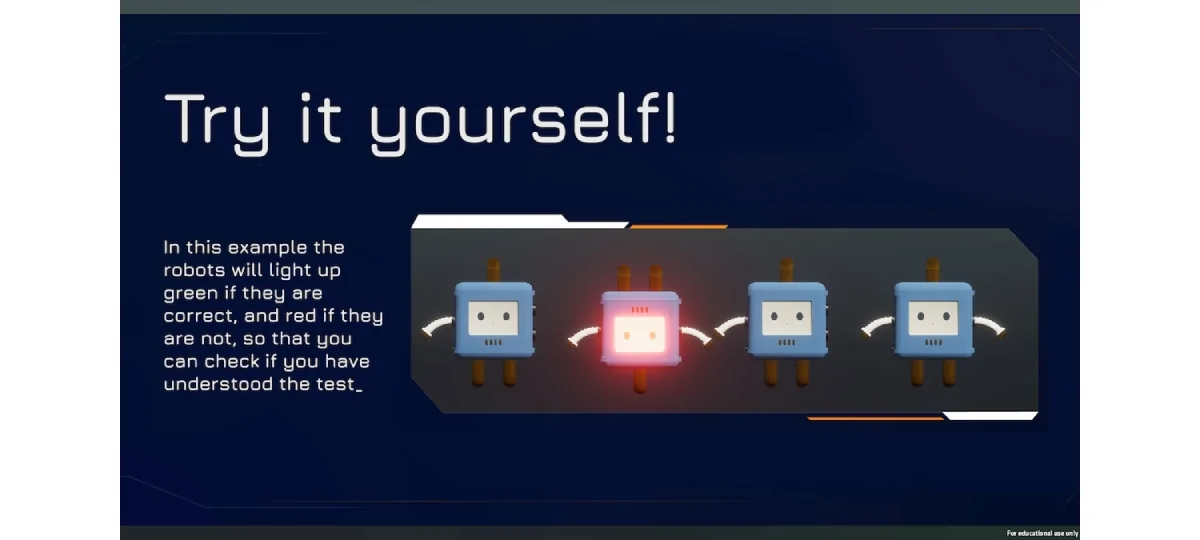
Attention Robots example: wrong robot marked.

Following the tutorial, a concise and informative example is presented to illustrate the game’s mechanics in a practical context, as shown in both Figure 6 and Figure 7. This example serves as a final preparatory step before the game officially begins. Progression to the actual game is subject to the psychologist’s assurance that the player comprehends the rules.

**Figure 8:**
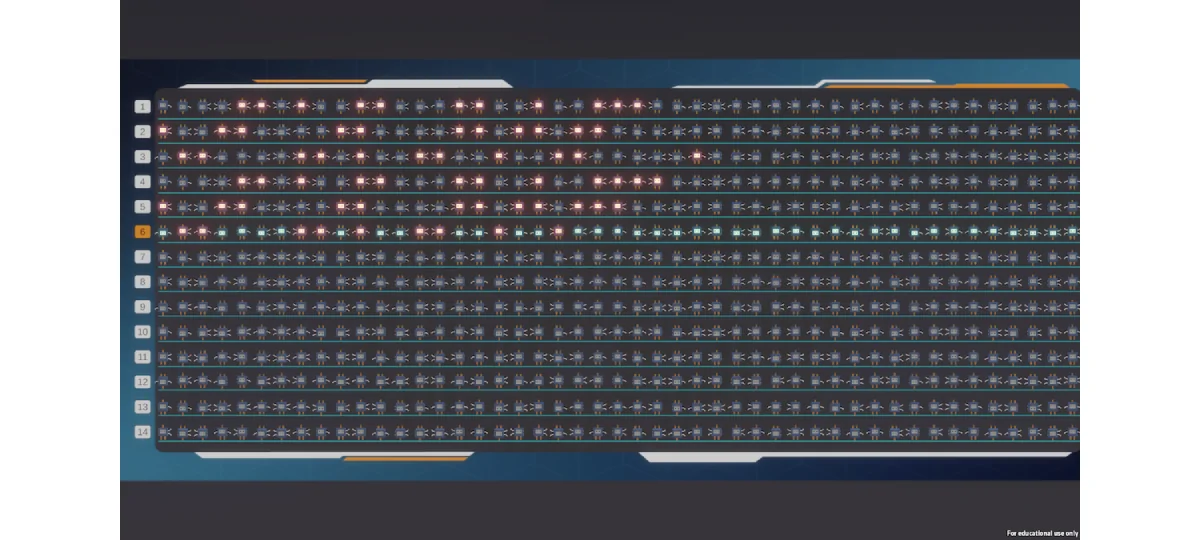
Attention Robots game screenshot.

During the game, players are asked to identify robots that adhere to the criteria outlined in the tutorial within a specific time frame of 20 seconds per line. The transition between lines is denoted by a distinct audio cue and the highlighting of the next line. The effect of this guidance on engagement or performance was not evaluated separately in this study. Correctly identified robots are marked in red for reference, and players can rectify mistakes by clicking on the robots again. The game concludes by presenting a clear differentiation between correctly and incorrectly marked robots. Figure 8 shows a screenshot of the game in progress.

The game recorded the following events and outputs: (1) hits, defined as correctly selected target robots; (2) omissions, defined as target robots that should have been selected but were not; (3) commissions, defined as nontarget robots selected incorrectly by the participant; (4) the last robot marked in each row, use to compute working speed (WS) and (5) where the gaze of the player was focused during the game.

From these recorded game events, additional metrics can be derived, including total commissions and total omissions.

#### Attention Robots Software and Hardware Architecture

**Figure 9:**
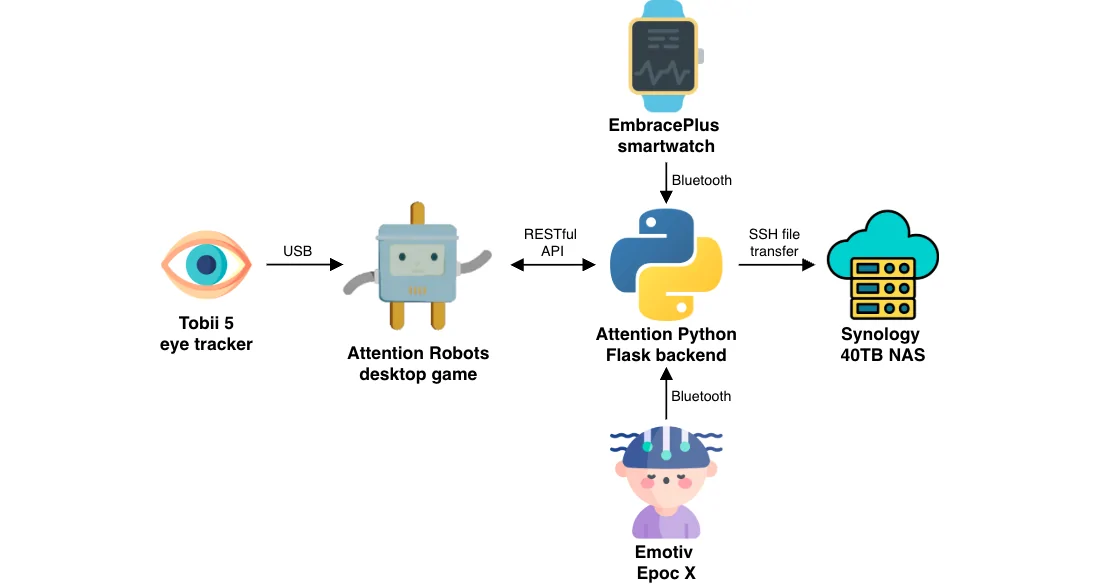
Software and hardware architecture of Attention Robots. API: application programming interface; NAS: network-attached storage; SSH: secure shell; USB: universal serial bus.

To facilitate data acquisition from multiple devices, a comprehensive software and hardware architecture, as depicted in Figure 9, has been implemented for Attention Robots within the broader Attention Game Series. The architecture separates the Unity game [33] from the data-management layer through a backend application program interface, allowing game events and behavioral outputs to be stored in a structured way independently of the game interface. In this study, this architecture is included only to document the technical context of Attention Robots; the validation analyses used the behavioral indicators reported above.

Although the platform was designed to be extensible to additional devices and future multimodal studies, those components were not analyzed in this manuscript and are not part of the evidence reported here. Therefore, the results should be interpreted as a behavioral cross-instrument validation of Attention Robots against the D2-R test. The broader multimodal data analysis will be conducted in an independent study using the dataset described in the *Scientific Data* data descriptor [34] and the data-capture system presented in this work, applying AI models to support ADHD screening research. Accordingly, this paper focuses on Attention Robots as a structured data-capture and behavioral assessment tool and on its preliminary correspondence with the D2-R test, rather than on analysis of the broader multimodal dataset.

#### The D2-R Test of Attention

The D2-R Test of Attention [31] is a paper-and-pencil test that involves various characters and symbols, in which the individual who takes part in the test is required to scan through a set of characters and mark specific ones that meet certain criteria. The test takes approximately 8 minutes to complete, and it has demonstrated its successful application in individuals aged 6 to 80 years [31].

The test consists of 14 lines with 57 items each. These lines contained the letters “d” and “p,” which could appear with 1 or 2 small dashes above or below. Participants have 20 seconds per line to carefully mark, from left to right, every letter “d” showing 2 dashes (both above, below, or one above and one below).

As described in [31], the following information is collected from the D2-R test: (1) WS, the sum of the last well-marked element in each row; (2) omissions, the sum of the omitted elements, when the participant skipped a target element (which should be marked); and (3) commissions, the sum of incorrectly marked elements, when the participant marked an element that should not be marked.

Two derived metrics were then calculated: concentration, computed as CN = WS – (O + C), and precision, computed as P = ([WS – CN]/WS)×100, where O denotes omissions and C denotes commissions.

Because concentration and precision are derived from WS, omissions, and commissions, they were interpreted as secondary composite indicators rather than independent primary outcomes.

This test showed good psychometric properties for its application through internal consistency Cronbach α values of 0.94 for general answer (the number of elements processed), 0.95 for correct guesses (the number of correctly marked target elements), 0.94 for omissions, and 0.93 for commissions [31].

### Measures and Covariates

The primary behavioral indicators were WS, omissions, and commissions, obtained from both Attention Robots and the D2-R test. Due to their derivation from WS, omissions, and commissions, concentration and precision were regarded as secondary composite indicators. Demographic variables included age, sex, and the original group variable assigned by the project psychologists. The group variable was used for descriptive reporting only, and the main validation analyses were conducted in the pooled sample. The current behavioral validation analyses did not incorporate the biometric and eye-tracking data that were gathered during the Attention Robots session for future multimodal study.

### Quality of Measurements and Instrumentation

The quality of the behavioral measurements was supported by a number of methods. Every participant was subject to the same task guidelines and timetable. In the Attention Robots session, participants completed a video tutorial and a practical example before starting the task, and the psychologist confirmed that the participant understood the rules. Before the assignment started, a computer technician was in charge of setting up and calibrating the recording devices and confirming that the system was operating correctly. The D2-R test was administered following its standardized instructions. The behavioral dataset used in these analyses contained complete values for all participants across the five indicators analyzed. Since the primary variables were derived from structured task outputs and D2-R scoring, no formal interrater reliability analysis was carried out.

Regarding instrumentation, Attention Robots was an ad hoc serious game–based assessment tool developed for this study within the Attention Game Series. It recorded hits, omissions, commissions, the last marked robot in each row, and gaze-related information. Only behavioral markers comparable to those of the D2-R test were examined in this paper. The D2-R Test of Attention, which has been shown to have psychometric qualities for assessing attention [31], served as the standard reference measure. As part of the larger data-capture platform, biometric devices were used during the Attention Robots session, but these recordings were not analyzed in this study.

Masking was not applied because participants and staff were aware of the assessment procedures, and the 2 instruments were clearly different in format.

### Evaluation Procedure and Data Collection

This section describes the comparative analysis between Attention Robots and the D2-R test [31], a standardized paper-and-pencil measure of attention.

Our goal was to examine potential correlations between the scores obtained from the Attention Robots test and those from the D2-R test. These analyses were intended to assess convergent validity and cross-instrument correspondence, not interchangeability, discriminative validity between ADHD and neurotypical participants, or stand-alone diagnostic use.

As noted earlier, the central objective of this section is to conduct an empirical evaluation and comparison of the Attention Robots test and the D2-R test. This evaluation was conducted through a controlled experiment, with its key features outlined in Textbox 1.

The experiments took place in 2 sessions of approximately 20 minutes, most of them carried out in the afternoons. The ISABIAL ethical committee confirmed that the Attention Robots test should always be performed in the first session and the D2-R test in the second session. Therefore, a crossover 2×2 experiment cannot be considered here since the approved experimental protocol must be followed in the specified sequence. The 2 sessions were not conducted on the same day. The second session took place at least on the following day and, depending on participant availability, could be scheduled later. Although scheduling was flexible, the protocol followed in each session was the same for all participants.

First, a comfortable environment was created, and demographic data were collected. Each participant was always accompanied by at least one psychologist and a computer technician. The computer technician was responsible for placing and calibrating the recording devices at the beginning of the session. The psychologist remained during the instruction phase to ensure that the participant understood the task rules. Once setup and instruction were completed, the task proceeded, while the staff remained available nearby only in case of technical issues, participant doubts, or difficulties in cooperation.

Afterward, the content and activities of the upcoming session were explained. Each session proceeded as follows:

- Session 1: Attention Robots test:
  The measuring elements (electroencephalography headset, EDA/heart rate wristband, and Tobii eye tracking) were placed and calibrated, and their proper operation was verified.
  Participants played the Attention Robots test on a high-end gaming computer.
  The following data were gathered: (1) the test indicators (WS, omissions, and commissions); (2) biometric data (EDA/heart rate); and (3) Tobii eye tracking data.
  The total duration was approximately 10 to 15 minutes.
- Session 2: D2-R test:
  The participants performed the standard D2-R test on a table using pencil and paper.
  The following information was gathered: the test indicators (WS, omissions, and commissions).
  The total duration was approximately 8 minutes.

After each session, participant data and outputs from both tests were stored in anonymized form. The stored records were identified only by study code, and the dataset used in these analyses was anonymized. Data were retained in the secure institutional storage infrastructure in accordance with the approved ethical and data-protection procedures.

The experimental sessions proceeded without any significant incidents affecting the behavioral assessment. A few younger participants expressed discomfort with wearing the electroencephalography headset. In these cases, electroencephalography acquisition was omitted, and the session continued without affecting the behavioral assessment.

**Main features of the experiment.**

**Null hypotheses**

- H_0A_: No correlation is found in the total number of the last well-marked elements (working speed) between the D2-R test and the Attention Robots test.
- H_0B_: No correlation is found in the omitted elements between the D2-R test and the Attention Robots test.
- H_0C_: No correlation is found in the incorrectly marked elements (commissions) between the D2-R test and the Attention Robots test.
- H_0D_: No correlation is found in the concentration between the D2-R test and the Attention Robots test.
- H_0E_: No correlation is found in the precision between the D2-R test and the Attention Robots test.

**Dependent variable**

- Correlation between attention test

**Independent variable**

- Use the D2-R Test of Attention.
- Use the Attention Robots test.

**Location**

- University Institute for Computing Research (Alicante, Spain)

**Date**

- From June 2022 to July 2023

**Participants**

- 58 participants aged 6 to 18 years

### Missing Data

The dataset contained complete Attention Robots and D2-R values for all 58 participants across WS, omissions, commissions, concentration, and precision. Thus, the proportion of missing data for the primary behavioral analyses was 0%, and missing completely at random testing and multiple imputation were not applicable. Biometric recordings were collected for future work and were not included in the current analyses; incomplete electroencephalography acquisition caused by headset discomfort did not affect inclusion in the behavioral dataset.

## Results

### Overview

In this section, the experiment results are presented. A total of 58 participants took part in the experiment. The mean age of the study participants was 13.4 (SD 3.90) years, comprising 24 female and 34 male individuals. The statistical significance was determined from the reported tests and corresponding *P* values.

### Experiment Results

Multimedia Appendix 1 contains the demographic variables and behavioral indicators obtained from the D2-R test and Attention Robots for each participant. Table 1 presents the descriptive statistics for both instruments, including maximum, minimum, mean, median, mode, and SD for the WS, omissions, commissions, concentration, and precision.

**Table 1:** Descriptive statistics for D2-R and Attention Robots tests.

	D2-R						Attention Robots				
	Working speed	Omissions	Commissions	Concentration	Precision	Working speed		Omissions	Commissions	Concentration	Precision
Sample, n	58	58	58	58	58	58		58	58	58	58
Minimum-maximum	27-247	0-50	0-13	21-239	0.6-35.7	92-439		0-15	0-21	69-436	0-25
Mean (SD)	139.276 (51.041)	8.621 (8.387)	3.707 (3.078)	126.828 (49.824)	9.747 (6.388)	262.328 (92.246)		5.172 (4.074)	3.362 (3.914)	253.793 (93.918)	4.062 (3.920)
Median (IQR)	134.5 (103.5-174.0)	6.0 (3.0-11.0)	3.0 (1.3-5.0)	121.0 (95.0-162.8)	8.2 (5.2-13.2)	258 (196.5-335.0)		4.5 (2.0-8.0)	2 (0.3-5.0)	252.5 (184.8-328.5)	3.2 (1.6-5.5)
Mode	188	6	2	100	8	274		1	0	182	3.6

### Correlation Analysis

The main analytic goal was to determine whether behavioral indicators from Attention Robots were correlated with corresponding indicators from the D2-R test.

First, we calculated the Spearman correlation coefficient (SCC) [35], which evaluates the monotonic relationship between 2 variables. In a monotonic relationship, the variables tend to change at the same time, but not necessarily at a constant rate [36], and are less sensitive to outliers. These calculations can be seen in Tables 2 and 3. Table 2 presents the SCC values, while Table 3 presents the *P* value matrix.

The SCC values represented in Table 2 can be interpreted as follows: values close to 1 indicate a strong positive monotonic relationship, meaning that as one variable increases, the other tends to increase; values close to –1 indicate a strong negative monotonic relationship, meaning that as one variable increases, the other tends to decrease; and values close to 0 suggest a lack of a monotonic relationship, meaning there is little or no association between the variables.

Table 4 summarizes the 5 prespecified cross-test SCC results with approximate 95% CIs.

**Table 2:** Spearman correlation coefficient matrix.

	AR^a^_WS^b^	AR_O^c^	AR_C^d^	AR_CN^e^	AR_P^f^	D2^g^_WS	D2_O	D2_C	D2_CN	D2_P
AR_WS	1.000	0.122	–0.610	0.997	–0.669	0.905	0.439	–0.406	0.921	–0.456
AR_O		1.000	–0.038	0.086	0.464	0.055	0.333	0.097	0.011	0.245
AR_C			1.000	–0.637	0.750	–0.590	–0.210	0.496	–0.615	0.423
AR_CN				1.000	–0.703	0.906	0.416	–0.481	0.926	–0.485
AR_P					1.000	–0.638	–0.090	0.549	–0.686	0.607
D2_WS						1.000	0.496	–0.415	0.978	–0.384
D2_O							1.000	–0.083	0.385	0.463
D2_C								1.000	–0.464	0.609
D2_CN									1.000	–0.511
D2_P										1.000

^a^AR: Attention Robots.

^b^WS: working speed.

^c^O: omissions.

^d^C: commissions.

^e^CN: concentration.

^f^P: precision.

^g^D2: D2-R Test of Attention.

**Table 3:** Spearman *P* value matrix.

	AR^a^_WS^b^	AR_O^c^	AR_C^d^	AR_CN^e^	AR_P^f^	D2^g^_WS	D2_O	D2_C	D2_CN	D2_P
AR_WS	—^h^	.36	<.001	<.001	<.001	<.001	.001	<.001	<.001	<.001
AR_O		—	.78	.52	<.001	.68	.01	.47	.94	.06
AR_C			—	<.001	<.001	<.001	.11	<.001	<.001	.001
AR_CN				—	<.001	<.001	.001	<.001	<.001	<.001
AR_P					—	<.001	.50	<.001	<.001	<.001
D2_WS						—	<.001	.001	<.001	.003
D2_O							—	.53	.003	<.001
D2_C								—	<.001	<.001
D2_CN									—	<.001

^a^AR: Attention Robots.

^b^WS: working speed.

^c^O: omissions.

^d^C: commissions.

^e^CN: concentration.

^f^P: precision.

^g^D2: D2-R Test of Attention.

^h^Not applicable.

**Table 4:** Spearman correlation results.

D2-R vs Attention Robots	Spearman coefficient (95% CI)^a^	*P* value
Working speed	0.905 (0.84-0.94)	<.001
Omissions	0.333 (0.08-0.54)	.01
Commissions	0.496 (0.27-0.67)	<.001
Concentration	0.926 (0.88-0.96)	<.001
Precision	0.607 (0.41-0.75)	<.001

^a^CIs were calculated using Fisher *z* transformation and are interpreted as precision estimates for the Spearman correlations.

Then, the Pearson correlation coefficient (PCC) was calculated [35]. This statistical metric measures the strength and direction of a linear relationship between 2 variables [37]. It quantifies the degree to which variations in one variable correspond with variations in another variable. It is sensitive to outliers. Table 5 presents the PCC values, and Table 6 presents the *P* value matrix.

The PCC values represented in Table 5 have the following interpretation: values close to 1 indicate a linear relationship, values close to –1 indicate a negative linear relationship, and values close to 0 indicate no linear relationship.

Table 7 summarizes the 5 prespecified cross-test PCC results with 95% CIs.

Therefore, based on the results obtained through the SCC and PCC, we can reject the null hypotheses H_0A_, H_0B_, H_0C_, H_0D_, and H_0E_. Accordingly, the analyses support statistically significant associations between the corresponding indicators obtained from the D2-R test and the Attention Robots test. Specifically, correlation is found between the D2-R test and the Attention Robots test in the total number of the last well-marked elements (WS), in the omitted elements, in the wrongly marked elements (commissions), and in concentration and precision.

These findings should not be interpreted as evidence that Attention Robots and the D2-R test are interchangeable or that Attention Robots can discriminate ADHD from neurotypical participants. They support preliminary convergent validity for corresponding behavioral indicators only.

**Table 5:** Pearson correlation coefficient matrix.

	AR^a^_WS^b^	AR_O^c^	AR_C^d^	AR_CN^e^	AR_P^f^	D2^g^_WS	D2_O	D2_C	D2_CN	D2_P
AR_WS	1.000	0.150	–0.546	0.998	–0.601	0.911	0.266	–0.414	0.916	–0.436
AR_O		1.000	–0.080	0.107	0.237	0.093	0.278	0.103	0.044	0.163
AR_C			1.000	–0.575	0.847	–0.568	–0.105	0.396	–0.587	0.493
AR_CN				1.000	–0.635	0.914	0.254	–0.427	0.922	–0.456
AR_P					1.000	–0.614	–0.068	0.367	–0.639	0.574
D2_WS						1.000	0.371	–0.364	0.986	–0.355
D2_O							1.000	–0.116	0.220	0.617
D2_C								1.000	–0.414	0.447
D2_CN									1.000	–0.493
D2_P										1.000

^a^AR: Attention Robots.

^b^WS: working speed.

^c^O: omissions.

^d^C: commissions.

^e^CN: concentration.

^f^P: precision.

^g^D2: D2-R Test of Attention.

**Table 6:** Pearson *P* value matrix.

	AR^a^_WS^b^	AR_O^c^	AR_C^d^	AR_CN^e^	AR_P^f^	D2^g^_WS	D2_O	D2_C	D2_CN	D2_P
AR_WS	—^h^	.26	<.001	<.001	<.001	<.001	.04	.001	<.001	.001
AR_O		—	.55	.42	.07	.49	.04	.44	.75	.22
AR_C			—	<.001	<.001	<.001	.43	.002	<.001	.001
AR_CN				—	<.001	<.001	.05	.001	<.001	<.001
AR_P					—	<.001	.61	.005	<.001	<.001
D2_WS						—	.004	.005	<.001	.003
D2_O							—	.43	.10	<.001
D2_C								—	.001	<.001
D2_CN									—	<.001

^a^AR: Attention Robots.

^b^WS: working speed.

^c^O: omissions.

^d^C: commissions.

^e^CN: concentration.

^f^P: precision.

^g^D2: D2-R Test of Attention.

^h^Not applicable.

**Table 7:** Pearson correlation results.

D2-R vs Attention Robots	Pearson coefficient (95% CI^a^)	*P* value
Working speed	0.911 (0.85-0.95)	<.001
Omissions	0.278 (0.02-0.50)	.04
Commissions	0.396 (0.15-0.59)	.002
Concentration	0.922 (0.87-0.95)	<.001
Precision	0.574 (0.37-0.72)	<.001

^a^CIs were calculated using Fisher *z* transformation and are interpreted as precision estimates for the Pearson correlations.

### Threats to the Experimental Validity

Validity threats were analyzed using the four categories described by [38]: conclusion, internal, external, and construct validity.

*Conclusion validity* pertains to factors that may lead to erroneous inferences about the observations [38]. In this study, 5 null hypotheses were tested using the PCC and SCC [36,37], yielding statistically significant *P* values at .05, thereby affirming our initial hypotheses with a considerable degree of certainty.

*Internal validity* concerns the design of the study, particularly whether the results are genuinely reflective of the data [38]. An important internal validity issue in this study was the fixed administration order, because Attention Robots was always administered before the D2-R according to the approved ethics protocol. This order could not be counterbalanced and may have introduced practice or fatigue effects [39]. In addition, although the procedure was standardized across participants, the interval between sessions depended on participant availability, which may have introduced additional variability. Additional internal validity considerations include the need for psychologist and technician support during device setup and instructional comprehension before the task began, as well as minor discomfort with the electroencephalography headset in some younger participants. The primary internal validity threat examined was instrumentation, as flawed tools could skew experimental outcomes.

To mitigate this, game development and experimental design were closely monitored by a team of neuropsychology experts. Additionally, the ethical committee of the ISABIAL approved the experiment. Participant attrition, or mortality, was addressed by scheduling sessions around participant availability, ensuring no dropouts. The sample should be interpreted as a feasibility/convenience sample for preliminary validation, and inference should be based on the reported correlation estimates, *P* values, and CIs [40].

*Construct validity* examines the degree to which the experimental setup and selected measures accurately represent the concept under investigation [38]. The D2-R variables, a well-established test for measuring attention [31], were used to avert validity issues. Additionally, Attention Robots underwent thorough testing by game developers and a pilot test prior to the experiment.

*External validity* relates to the generalizability of the results [38]. The participant age range was 6 to 18 years, which allowed the initial validation to cover the intended target population. However, developmental differences across this span may influence absolute performance levels, so these findings should be interpreted as evidence of overall cross-instrument association across a heterogeneous age range rather than age-specific performance equivalence. In addition, recruitment differed between groups: participants with ADHD were referred from a specialized clinical unit, whereas healthy control participants were recruited through personal contacts and recommendations. This difference may introduce selection bias and limit generalizability. Although both neurotypical individuals and people with ADHD participated, no differentiation was made in the analysis. Future studies should include age-stratified results, age-adjusted models, and analyses by group and clinical representation.

## Discussion

### Principal Findings

The primary objective of this study was to examine whether the behavioral indicators obtained from the Attention Robots test corresponded with the same indicators obtained from the D2-R test [31]. The results supported the 5 prespecified correlation hypotheses and provided preliminary evidence of convergent validity [41] for Attention Robots as a complementary behavioral attention-assessment format. These findings should be interpreted as evidence of correspondence between both tests, not as evidence that Attention Robots can replace the D2-R test or be used as a stand-alone diagnostic instrument.

### Interpretation

The results are consistent with the design of Attention Robots. The game is based on a timed visual-search task in which participants must identify target robots according to specific rules. This structure preserves several demands of the D2-R test, including sustained scanning, selective attention, inhibitory control, and a speed-accuracy balance [31]. The strongest correspondence was observed for WS, while the derived concentration measure showed similarly high associations. Because concentration is calculated from WS, omissions, and commissions, it should be interpreted as a composite indicator rather than as an independent source of evidence. One possible explanation is that error-related indicators may be more sensitive to differences between both formats, such as stimulus design, screen layout, audiovisual guidance, and response modality [42].

A complementary explanation lies in each indicator. WS summarizes the amount of material processed within the time limit, a production measure that is largely preserved when the task moves from paper to screen. In contrast, omissions and commissions are low-frequency events that capture lapses of sustained attention and failures of inhibitory control [24,25], so a small number of discrepant responses can shift their totals considerably, and their skewed, nonnormal distributions can affect the size of correlation estimates [37]. This pattern is also consistent with the heterogeneity of executive functioning described in ADHD populations, where speed-related and inhibition-related performance do not necessarily covary within the same individual [3,9].

The convergence between the 2 correlation methods strengthens this interpretation. SCC and PCC yielded the same qualitative pattern, with the strongest associations for WS and concentration, and an intermediate association for precision. The weakest associations were observed for commissions and omissions. These results suggest that the observed relationships are monotonic and approximately linear. They are also not driven by a small number of extreme scores [36,37]. This correspondence is also coherent with the broader finding that computerized adaptations of attention tasks tend to preserve the constructs measured by their traditional counterparts while introducing format-specific variance [8,16]. Within this framework, the strong association for WS indicates that the game preserves the temporal and perceptual demands of the D2-R. The weaker associations for omissions and commissions most likely reflect the surface differences between formats, as described above. However, the present design cannot fully separate construct differences from format effects.

There are a number of limitations to this study. First, the sample was based on convenience recruitment, and no a priori power analysis was performed. Therefore, the results should be considered preliminary [40]. Second, the analyses were carried out in the pooled sample, and the age range was wide. This limits conclusions about age-specific performance. Third, ADHD and neurotypical participants were not analyzed separately, so the study does not evaluate discriminative validity or diagnostic classification. Fourth, Attention Robots was always administered before the D2-R test, following the approved ethical protocol, so order effects cannot be ruled out. Fifth, no agreement analysis was performed, so the results do not show whether scores from both tests can be used interchangeably [41]. Lastly, although biometric information was gathered during the Attention Robots session, it was not examined in this paper.

### Similarity of Results

These findings are also aligned with previous studies showing that computerized tasks, virtual environments, and serious games can be used to collect behavioral indicators related to attention and executive functioning [8,30]. However, many serious game studies focus primarily on feasibility, engagement, training effects, or diagnostic classification, rather than on direct comparison with standardized tests. This study contributes to this literature by directly comparing game-derived behavioral indicators with corresponding indicators from an established attention test. This comparison helps clarify which aspects of game performance closely align with paper-and-pencil measures and which may instead reflect task-specific features of the digital environment. However, this study only analyzed behavioral data. Although Attention Robots was designed as a child-oriented serious game, user-experience outcomes such as enjoyment, immersion, acceptability, and preference were not measured. Therefore, this study cannot conclude that Attention Robots is more engaging or more acceptable than traditional paper-and-pencil assessments.

Placing these results next to specific prior efforts clarifies this contribution. Virtual reality continuous performance tests have been compared with their traditional counterparts at the level of overall task performance [8,20], and driving or runner video games have been related to symptom measures and diagnostic status in ADHD [18,23]. Serious games have also been studied mainly as training or rehabilitation tools, with systematic reviews focusing on symptom improvement and cognitive outcomes rather than on measurement validity [11,30]. In contrast, this study compared each behavioral indicator of the game directly with the corresponding indicator of a normed attention test [31], which allows the correspondence to be examined separately for speed, error, and composite measures.

The observed pattern of correspondence is also consistent with this previous work. Validation studies of computerized and virtual reality attention tasks typically find that the strength of association with traditional instruments varies across indicators and that part of the variance is attributable to format-specific factors rather than to the measured construct [8,16]. Within the continuous performance test tradition, omissions and commissions are likewise treated as related but separable indicators of inattention and impulsivity [24,25], so their weaker cross-format correspondence in this study is not unexpected. These results extend this literature to a robot-themed cancellation game for children and adolescents, showing that the strongest correspondence occurs for speed-based and composite indicators and the weakest for infrequent error events.

### Generalizability

Our findings may be cautiously generalized to the population and conditions that the study was designed to represent. The sample covered the intended age range of 6 to 18 years, and the anchor instrument, the D2-R, is validated for individuals from 6 to 80 years of age [31]. Sessions were short, structured, and supervised, and took place in a comfortable environment. A psychologist verified that each participant understood the task, in line with how standardized attention tests are administered in practice [31]. Since the behavioral indicators are automatically recorded by the game itself, this component of the platform could in principle be used in clinical or research settings with similar technical resources.

At the same time, several characteristics of the sample constrain generalizability. Recruitment was based on convenience sampling [38]. Participants with ADHD were referred from a specialized clinical unit, and control participants were recruited through personal contacts and recommendations, which may introduce selection bias and limit the representativeness of both groups. Moreover, the analyses were conducted on the pooled sample across a wide age range, so the results describe the overall association between instruments rather than age-specific or group-specific performance. In addition, the 2 groups differed in sex composition, so group and sex are confounded in this sample, and neither can be interpreted independently of the other. Finally, data were collected in a single geographic and institutional context and under a fixed administration order, so site-related effects and order-related effects, such as practice or fatigue, cannot be excluded [38].

Within these constraints, what generalizes most defensibly is the association between corresponding indicators, not the interchangeability of individual scores. The game preserves the core attentional demands of a validated cancellation test [31], and research on computerized and virtual reality assessment indicates that well-designed digital formats can retain the constructs of the instruments they adapt [8,16]. Extending these findings to individual clinical interpretation in real-world practice would additionally require age- and group-stratified norms, agreement analyses between formats, and replication in larger multisite samples with balanced recruitment.

### Implications

The results should also be interpreted in the broader context of ADHD assessment. ADHD diagnosis requires information from different sources and settings, including clinical interviews, family and teacher reports, and structured assessments [7]. For this reason, Attention Robots should be considered a complementary tool for collecting objective behavioral data, not a diagnostic tool by itself. Clinical guidelines state that ADHD cannot be established from a single observation: the symptoms must be shown to occur in more than one setting, such as home and school, and the information must come from several people who know the child, typically parents and teachers [6,22]. This information is usually gathered with rating scales, but their quality depends on which of these adults are available and on how each of them perceives the child’s behavior, which is inevitably subjective. A task such as Attention Robots complements these scales because it records the child’s actual performance automatically, adding objective attention-related data. This need for objective measures that do not depend on an external observer is one of the main reasons behind the growing interest in computerized approaches to ADHD assessment [5,14].

The practical relevance of this approach lies in its ability to automatically and consistently record performance through an interface that may be more accessible to children and adolescents than traditional formats. Since hits, omissions, commissions, and the last marked element are stored as discrete events, scoring requires no manual correction of a paper sheet, and the full response record remains available for later inspection. Realizing this benefit in individual clinical work would nevertheless require age- and group-stratified norms and cutoffs, which the present pooled design was not intended to produce. A realistic development path is illustrated by computerized continuous performance tests, which became clinically usable once normative data and standardized scoring were established [24]. Because Attention Robots stores every response as a discrete event, the same records that supported this validation could also support longitudinal monitoring, in which repeated sessions of the same child are compared over time, although such use would first require evidence of test-retest reliability [24], which this study does not provide.

The findings also matter for the multimodal research that the platform was built to support. Attention Robots is the data-acquisition layer of a system that records data from various devices during the same session. By establishing that 5 of its behavioral indicators align with those of a standardized instrument, this study provides a reference for the multimodal dataset released separately [34]. This interpretation is also consistent with recent research on AI-based and multimodal approaches for ADHD assessment, where behavioral and biometric data are considered promising but still require further validation before clinical use [14,29].

Finally, the results have implications for the design of digital assessment instruments. Rather than creating a new task and studying it in isolation, this work adapted the structure of an existing validated test into a game format and then measured how much of the original measurement behavior was preserved. Previous game-based approaches to ADHD assessment have generally been developed as novel tasks that are subsequently related to symptom measures or diagnostic status [18,23], and serious games more broadly have been evaluated mainly as interventions [11,30]. The present approach, in which each game indicator has a direct counterpart in a normed test [31], follows the same logic as adaptations of established instruments to virtual environments [20] and offers a replicable strategy for building child-oriented digital assessments whose outputs remain psychometrically interpretable.

### Conclusions

Attention assessment in ADHD still relies largely on clinical interviews and rating scales based on subjective observation. Although standardized tests such as the D2-R provide objective measurements, their paper-and-pencil format may be less appealing to children and does not use interactive digital environments. Serious games have been proposed as a more engaging means of capturing attention-related behavior, but most have been validated against symptom scales or diagnostic status rather than an established, normed attention test. This study addressed this gap by developing Attention Robots, a serious game that reproduces the attentional demands of the D2-R, and examining the convergence between the behavioral indicators obtained with both instruments in children and adolescents. The findings provide preliminary evidence of convergent validity for selected attention-related behavioral indicators, with stronger convergence for WS and concentration than for error-related indicators. The game combines robot-based stimuli, dynamic audiovisual guidance, and structured behavioral data collection in a child-oriented environment and, unlike previous digital and serious game approaches, it was directly compared with an established standardized attention test [30,31]. Nevertheless, the current evidence is preliminary, is limited to behavioral indicators, and should not be interpreted as demonstrating diagnostic validity.

In real-world settings, Attention Robots could be developed further as a complementary component of multimethod ADHD assessment. Realizing this potential will require a staged validation process in which the indicator-level correspondence demonstrated in this study is followed by additional evaluation before the tool can be considered for applied use. Accordingly, these findings position Attention Robots as a structured source of objective attention-related behavioral data and as a foundation for the multimodal research supported by the platform. Future studies should use larger and more appropriately stratified samples, report demographic characteristics separately for each group, examine age-adjusted and group-specific models, evaluate agreement between scores [41] and diagnostic performance, and directly assess user experience. They should also investigate the broader multimodal data collected through the platform to determine whether combining behavioral, eye-tracking, electroencephalography, and physiological measures provides useful information for ADHD-related screening and assessment research [34].

## Appendix

Multimedia Appendix 1 Experiment data.

## Abbreviations

ADHD: attention-deficit/hyperactivity disorder
DSM-5: Diagnostic and Statistical Manual of Mental Disorders, Fifth Edition
EDA: electrodermal activity
ISABIAL: Alicante Institute for Health and Biomedical Research
PCC: Pearson correlation coefficient
SCC: Spearman correlation coefficient
WS: working speed

## Data Availability

Multimedia Appendix 1 contains the behavioral metrics and demographic variables used in these validation analyses. Raw biometric recordings (electroencephalography, electrodermal activity/heart rate, and eye-tracking) were collected for future multimodal research and are not publicly released as part of this paper.

## References

[1] Faraone SV, Larsson H (2019). Genetics of attention deficit hyperactivity disorder. Mol Psychiatry.

[2] Cheng N, Bryce S, Takagi M, Pert A, Rattray A, Fisher E, Lai M, Geljic M, Youn S, Wood SJ, Allott K (2025). The prevalence of attention deficit hyperactivity disorder in psychotic disorders: systematic review and meta-analysis. Schizophr Bull.

[3] Willcutt EG, Doyle AE, Nigg JT, Faraone SV, Pennington BF (2005). Validity of the executive function theory of attention-deficit/hyperactivity disorder: a meta-analytic review. Biol Psychiatry.

[4] Wong T, Chang Y, Wang M, Chang Y (2023). The effectiveness of child-centered play therapy for executive functions in children with attention-deficit/hyperactivity disorder. Clin Child Psychol Psychiatry.

[5] Loh HW, Ooi CP, Barua PD, Palmer EE, Molinari F, Acharya UR (2022). Automated detection of ADHD: current trends and future perspective. Comput Biol Med.

[6] American Psychiatric Association (2013). Diagnostic and Statistical Manual of Mental Disorders: DSM-5.

[7] Canu WH, Eddy LD (2015). Attention-deficit hyperactivity disorder: a handbook for diagnosis and treatment. Cogn Behav Ther.

[8] Parsons TD, Duffield T, Asbee J (2019). A comparison of virtual reality classroom continuous performance tests to traditional continuous performance tests in delineating ADHD: a meta-analysis. Neuropsychol Rev.

[9] Kofler M, Irwin L, Soto E, Groves NB, Harmon SL, Sarver DE (2019). Executive functioning heterogeneity in pediatric ADHD. J Abnorm Child Psychol.

[10] Martin-Moratinos M, Bella-Fernández M, Rodrigo-Yanguas M, González-Tardón C, Li C, Wang P, Royuela A, Lopez-Garcia P, Blasco-Fontecilla H (2025). Effectiveness of a virtual reality serious video game (The Secret Trail of Moon) for emotional regulation in children with attention-deficit/hyperactivity disorder: randomized clinical trial. JMIR Serious Games.

[11] Doulou A, Pergantis P, Drigas A, Skianis C (2025). Managing ADHD symptoms in children through the use of various technology-driven serious games: a systematic review. Multimodal Technol Interact.

[12] Alabdulakareem E, Jamjoom M (2020). Computer-assisted learning for improving ADHD individuals’ executive functions through gamified interventions: a review. Entertain Comput.

[13] Teruel MA, Navarro E, Romero D (2017). An innovative tool to create neurofeedback games for ADHD treatment. Natural and Artificial Computation for Biomedicine and Neuroscience.

[14] Zaheer A, Akhtar A (2025). Artificial intelligence as a support to diagnose ADHD: an insight of unorthodox approaches: a scoping review. Child Neuropsychol.

[15] Guigou Y, Hennequin A, Marchand T (2025). Preliminary results of the EPIDIA4Kids study on brain function in children: multidimensional ADHD-related symptomatology screening using multimodality biometry. Front Psychiatry.

[16] Neguț A, Matu S, Sava FA, David D (2016). Virtual reality measures in neuropsychological assessment: a meta-analytic review. Clin Neuropsychol.

[17] Chaytor N, Schmitter-Edgecombe M, Burr R (2006). Improving the ecological validity of executive functioning assessment. Arch Clin Neuropsychol.

[18] Sujar A, Bayona S, Delgado-Gómez D, Miguélez-Fernández C, Ardoy-Cuadros J, Peñuelas-Calvo I, Baca-García E, Blasco-Fontecilla H (2022). Attention deficit hyperactivity disorder assessment based on patient behavior exhibited in a car video game: a pilot study. Brain Sci.

[19] Siemerkus J, Irle E, Schmidt-Samoa C, Dechent P, Weniger G (2012). Egocentric spatial learning in schizophrenia investigated with functional magnetic resonance imaging. Neuroimage Clin.

[20] Parsons TD, Courtney CG (2014). An initial validation of the virtual reality paced auditory serial addition test in a college sample. J Neurosci Methods.

[21] Hassan A, Pinkwart N, Shafi M (2021). Serious games to improve social and emotional intelligence in children with autism. Entertain Comput.

[22] Faraone SV, Asherson P, Banaschewski T, Biederman J, Buitelaar JK, Ramos-Quiroga JA, Rohde LA, Sonuga-Barke EJS, Tannock R, Franke B (2015). Attention-deficit/hyperactivity disorder. Nat Rev Dis Primers.

[23] Delgado-Gómez D, Sújar A, Ardoy-Cuadros J, Bejarano-Gómez A, Aguado D, Miguelez-Fernandez C, Blasco-Fontecilla H, Peñuelas-Calvo I (2020). Objective assessment of attention-deficit hyperactivity disorder (ADHD) using an infinite runner-based computer game: a pilot study. Brain Sci.

[24] Conners CK, Staff MHS, Connelly V (2000). Conners' Continuous Performance Test II (CPT II v. 5).

[25] Trommer BL, Hoeppner JA, Lorber R, Armstrong KJ (1988). The go-no-go paradigm in attention deficit disorder. Ann Neurol.

[26] Moradi N, Rajabi S, Mansouri Nejad A (2024). The effect of neurofeedback training combined with computer cognitive games on the time perception, attention, and working memory in children with ADHD. Appl Neuropsychol Child.

[27] Tamm L, Epstein JN, Peugh JL, Nakonezny PA, Hughes CW (2013). Preliminary data suggesting the efficacy of attention training for school-aged children with ADHD. Dev Cogn Neurosci.

[28] Snyder HR, Miyake A, Hankin BL (2015). Advancing understanding of executive function impairments and psychopathology: bridging the gap between clinical and cognitive approaches. Front Psychol.

[29] Ul Ain Q, Jawed S, Rauf Subhani A, Haider Butt B, Usman Akram M (2025). Examining AI-powered ADHD diagnosis: current trends, key challenges, and future directions in the field. IEEE Access.

[30] Shahmoradi L, Mohammadian F, Rahmani Katigari M (2022). A systematic review on serious games in attention rehabilitation and their effects. Behav Neurol.

[31] Brickenkamp R, Schmidt-Atzert L, Liepmann D (2022). D2-R. Revised Attention Test Assessment of Selective Attention and Concentration (1st ed).

[32] Teruel M.A, Trujillo J, Maté A, Ferrer-Cascales R, Albaladejo-Blázquez N, Ruiz-Robledillo N (2023). Attention Robots source code. University of Alicante Institutional Repository (RUA).

[33] (2023). Unity Technologies.

[34] Trujillo J, Ferrer-Cascales R, Teruel MA, Ruiz-Robledillo N, Sanchis J, García-Ponsoda Sandra, Panagiotidis-Arrizabalaga A, Albaladejo-Blázquez Natalia, Martínez-Nicolás Ángela, García-Carrasco Jorge, Reina A, Lavalle A, Maté Alejandro, Costa-López Borja (2026). A multimodal dataset for neurophysiological and AI applications. Sci Data.

[35] Smeeton N, Spencer N, Sprent P (2025). Applied Nonparametric Statistical Methods.

[36] Hauke J, Kossowski T (2011). Comparison of values of Pearson's and Spearman's correlation coefficients on the same sets of data. Quaestiones Geographicae.

[37] de Winter JCF, Gosling SD, Potter J (2016). Comparing the Pearson and Spearman correlation coefficients across distributions and sample sizes: a tutorial using simulations and empirical data. Psychol Methods.

[38] Wohlin C, Wesslén A, Regnell B (2012). Experimentation in Software Engineering.

[39] Calamia M, Markon K, Tranel D (2012). Scoring higher the second time around: meta-analyses of practice effects in neuropsychological assessment. Clin Neuropsychol.

[40] Kukull WA, Ganguli M (2012). Generalizability: the trees, the forest, and the low-hanging fruit. Neurology.

[41] Bland JM, Altman DG (2010). Statistical methods for assessing agreement between two methods of clinical measurement. Int J Nurs Stud.

[42] Bauer RM, Iverson GL, Cernich AN, Binder LM, Ruff RM, Naugle RI (2012). Computerized neuropsychological assessment devices: joint position paper of the American Academy of Clinical Neuropsychology and the National Academy of Neuropsychology. Arch Clin Neuropsychol.

