# Supplementary material for: Serious Game–Based Attention Assessment for Attention-Deficit/Hyperactivity Disorder in Children and Adolescents: Preliminary Cross-Sectional Convergent Validation Study
Source: JMIR Serious Games. 2026 Aug 28;14:e80441. doi: 10.2196/80441 (PMC13570240; doi:10.2196/80441)
Supplement: Supplementary file 1 [file games_v14i1e80441_app1.docx]

# Gamifying Attention Assessment for Attention Deficit Hyperactivity Disorder

## Supplementary Information 1

Supplementary Table 1 - Experiment data

| **id** | **group** | **sex** | **age** | **AR_WS** | **AR_O** | **AR_C** | **AR_CN** | **AR_P** | **D2_WS** | **D2_O** | **D2_C** | **D2_CN** | **D2_P** |
| --- | --- | --- | --- | --- | --- | --- | --- | --- | --- | --- | --- | --- | --- |
| 1 | 2 | 2 | 11 | 274 | 9 | 1 | 264 | 3.6 | 162 | 6 | 7 | 149 | 8.0 |
| 2 | 2 | 1 | 8 | 138 | 10 | 3 | 125 | 9.4 | 94 | 9 | 6 | 79 | 16.0 |
| 3 | 1 | 1 | 10 | 213 | 1 | 2 | 210 | 1.4 | 106 | 6 | 5 | 95 | 10.4 |
| 4 | 2 | 2 | 10 | 194 | 7 | 5 | 182 | 6.2 | 75 | 3 | 3 | 69 | 8.0 |
| 5 | 1 | 1 | 7 | 92 | 2 | 21 | 69 | 25.0 | 27 | 2 | 4 | 21 | 22.2 |
| 6 | 1 | 1 | 17 | 335 | 1 | 2 | 332 | 0.9 | 171 | 2 | 2 | 167 | 2.3 |
| 7 | 1 | 1 | 14 | 212 | 4 | 2 | 206 | 2.8 | 120 | 1 | 0 | 114 | 5.0 |
| 8 | 1 | 1 | 10 | 182 | 12 | 2 | 168 | 7.7 | 74 | 2 | 7 | 65 | 12.2 |
| 9 | 1 | 1 | 10 | 137 | 2 | 6 | 129 | 5.8 | 135 | 2 | 9 | 124 | 8.1 |
| 10 | 2 | 1 | 18 | 347 | 0 | 0 | 347 | 0.0 | 191 | 7 | 1 | 183 | 4.2 |
| 11 | 2 | 2 | 18 | 394 | 13 | 0 | 381 | 3.3 | 228 | 22 | 5 | 201 | 11.8 |
| 12 | 2 | 1 | 18 | 430 | 10 | 0 | 420 | 2.3 | 217 | 12 | 2 | 203 | 6.5 |
| 13 | 2 | 1 | 18 | 368 | 1 | 0 | 367 | 0.3 | 182 | 11 | 0 | 171 | 6.0 |
| 14 | 2 | 1 | 18 | 397 | 8 | 0 | 389 | 2.0 | 214 | 22 | 0 | 192 | 10.3 |
| 15 | 2 | 1 | 18 | 382 | 7 | 0 | 375 | 1.8 | 227 | 18 | 0 | 209 | 7.9 |
| 16 | 2 | 2 | 18 | 439 | 3 | 0 | 436 | 0.7 | 188 | 11 | 4 | 173 | 8.0 |
| 17 | 2 | 1 | 18 | 249 | 8 | 1 | 240 | 3.6 | 121 | 10 | 3 | 108 | 10.7 |
| 18 | 2 | 1 | 18 | 344 | 1 | 0 | 343 | 0.3 | 175 | 0 | 1 | 174 | 0.6 |
| 19 | 2 | 2 | 18 | 378 | 5 | 0 | 373 | 1.3 | 247 | 8 | 0 | 239 | 3.2 |
| 20 | 2 | 2 | 18 | 300 | 1 | 1 | 298 | 0.7 | 160 | 6 | 0 | 154 | 3.8 |
| 21 | 2 | 1 | 18 | 335 | 7 | 1 | 327 | 2.4 | 188 | 21 | 2 | 165 | 12.2 |
| 22 | 2 | 1 | 18 | 438 | 2 | 0 | 436 | 0.5 | 234 | 4 | 0 | 230 | 1.7 |
| 23 | 2 | 2 | 18 | 274 | 0 | 0 | 274 | 0.0 | 180 | 3 | 0 | 177 | 1.7 |
| 24 | 2 | 1 | 18 | 350 | 6 | 0 | 344 | 1.7 | 180 | 20 | 4 | 156 | 13.3 |
| 25 | 2 | 2 | 13 | 259 | 3 | 2 | 254 | 1.9 | 126 | 7 | 1 | 118 | 6.3 |
| 26 | 2 | 1 | 11 | 291 | 14 | 5 | 272 | 6.5 | 151 | 10 | 5 | 136 | 9.9 |
| 27 | 2 | 2 | 14 | 215 | 2 | 7 | 206 | 4.2 | 132 | 1 | 6 | 125 | 5.3 |
| 28 | 2 | 2 | 18 | 422 | 10 | 2 | 410 | 2.8 | 241 | 11 | 2 | 228 | 5.4 |
| 29 | 2 | 2 | 13 | 336 | 4 | 3 | 329 | 2.1 | 156 | 17 | 3 | 136 | 12.8 |
| 30 | 2 | 2 | 13 | 161 | 5 | 2 | 154 | 4.3 | 125 | 17 | 5 | 103 | 17.6 |
| 31 | 2 | 2 | 10 | 140 | 1 | 1 | 138 | 1.4 | 68 | 0 | 3 | 63 | 7.4 |
| 32 | 2 | 2 | 15 | 321 | 5 | 3 | 313 | 2.5 | 161 | 6 | 2 | 153 | 5.0 |
| 33 | 1 | 1 | 16 | 291 | 7 | 2 | 282 | 3.1 | 103 | 1 | 2 | 100 | 2.9 |
| 34 | 2 | 2 | 10 | 193 | 9 | 4 | 180 | 6.7 | 143 | 50 | 1 | 92 | 35.7 |
| 35 | 1 | 1 | 15 | 377 | 0 | 6 | 371 | 1.6 | 200 | 7 | 6 | 187 | 6.5 |
| 36 | 2 | 2 | 8 | 198 | 3 | 2 | 193 | 2.5 | 97 | 8 | 5 | 84 | 13.4 |
| 37 | 2 | 1 | 8 | 133 | 2 | 3 | 128 | 3.8 | 78 | 5 | 5 | 68 | 12.8 |
| 38 | 1 | 1 | 15 | 257 | 1 | 0 | 256 | 0.4 | 134 | 5 | 2 | 127 | 5.2 |
| 39 | 2 | 2 | 11 | 196 | 7 | 7 | 182 | 7.1 | 119 | 11 | 5 | 103 | 13.4 |
| 40 | 2 | 1 | 7 | 125 | 0 | 4 | 121 | 3.2 | 69 | 1 | 2 | 66 | 4.3 |
| 41 | 1 | 1 | 6 | 117 | 2 | 8 | 107 | 8.5 | 73 | 6 | 4 | 63 | 13.7 |
| 42 | 2 | 2 | 9 | 93 | 0 | 11 | 82 | 11.8 | 50 | 4 | 9 | 37 | 26.0 |
| 43 | 1 | 1 | 14 | 282 | 4 | 11 | 267 | 5.3 | 150 | 27 | 6 | 117 | 22.0 |
| 44 | 1 | 1 | 14 | 261 | 8 | 2 | 251 | 3.8 | 140 | 15 | 0 | 125 | 10.7 |
| 45 | 1 | 1 | 11 | 271 | 2 | 2 | 267 | 1.5 | 141 | 10 | 2 | 129 | 8.5 |
| 46 | 2 | 2 | 9 | 226 | 15 | 7 | 204 | 9.7 | 122 | 6 | 11 | 105 | 13.9 |
| 47 | 1 | 2 | 14 | 242 | 9 | 0 | 233 | 3.7 | 117 | 7 | 4 | 106 | 9.4 |
| 48 | 1 | 2 | 16 | 311 | 7 | 3 | 301 | 3.2 | 154 | 10 | 1 | 143 | 7.1 |
| 49 | 2 | 2 | 12 | 256 | 11 | 3 | 242 | 5.5 | 103 | 0 | 3 | 100 | 2.9 |
| 50 | 2 | 1 | 9 | 227 | 4 | 7 | 216 | 4.8 | 123 | 2 | 7 | 114 | 7.3 |
| 51 | 2 | 2 | 11 | 184 | 1 | 11 | 172 | 6.5 | 102 | 6 | 9 | 87 | 14.7 |
| 52 | 1 | 1 | 10 | 240 | 13 | 11 | 216 | 10.0 | 80 | 7 | 4 | 69 | 13.8 |
| 53 | 2 | 1 | 16 | 275 | 1 | 3 | 271 | 1.5 | 154 | 14 | 13 | 127 | 17.5 |
| 54 | 1 | 1 | 7 | 125 | 2 | 6 | 117 | 6.4 | 73 | 2 | 4 | 67 | 8.2 |
| 55 | 1 | 1 | 16 | 255 | 5 | 5 | 245 | 3.9 | 115 | 6 | 0 | 109 | 5.2 |
| 56 | 2 | 1 | 9 | 225 | 8 | 0 | 217 | 3.6 | 116 | 6 | 10 | 100 | 13.8 |
| 57 | 1 | 1 | 12 | 224 | 7 | 4 | 213 | 4.9 | 105 | 4 | 6 | 95 | 9.5 |
| 58 | 2 | 2 | 18 | 284 | 8 | 1 | 275 | 3.2 | 161 | 3 | 2 | 156 | 3.1 |
